# The Puzzle of the New Type of Intermediate in the Course of [2 + 2] Cycloaddition with the Participation of Conjugated Nitroalkenes: MEDT Computational Study

**DOI:** 10.3390/molecules30112410

**Published:** 2025-05-30

**Authors:** Radomir Jasiński, Agnieszka Kącka-Zych

**Affiliations:** Department of Organic Chemistry and Technology, Cracow University of Technology, Warszawska 24, 31-155 Cracow, Poland

**Keywords:** [2 + 2] cycloaddition, nitroalkenes, stereochemistry, pseudoradical intermediate, DFT calculations, mechanism, molecular electron density theory

## Abstract

The phenomena of regio- and stereoselectivity and the molecular mechanism of the [2 + 2] cycloaddition reaction between (E)-2-arylnitroethenes and the ynamine molecular system were analyzed using wb97xd/6-311 + G(d) (PCM) quantumchemical calculations. It was found that, independently of the stepwise nature of the cycloaddition, the full retention of the stereoconfiguration of the nitroalkene can be interpreted and explained. Next, the analysis of the electronic properties of the localized reaction intermediate suggests its possible zwitterionic nature. Additionally, the solvent and the substituent effect on the reaction course were also evaluated. In consequence, the proposed mechanism can be treated as general for some groups of [2 + 2] cycloaddition processes. Lastly, for the model process, the full Bonding Evolution Theory (BET) analysis along the reaction coordinate was performed. It was found that the [2 + 2] cycloaddition reaction between (E)-2-phenylonitroethene and ynamine begins with the formation of two *pseudoradical* centers at the C2 and C3 atoms. First, a C2-C3 single bond is formed in phase V by combining two *pseudoradical* centers, while the formation of a second C4-C1 single bond begins at the last, eleventh phase of the reaction path. A BET analysis of intermediate (**I**) allows it to be classified as a compound with a *pseudoradical* structure. Next to zwitterions and biradicals, it is evidently new type of intermediate on the path of the [2 + 2] cycloaddition reaction.

## 1. Introduction

Cycloaddition reactions are most universal and selective methodology for the preparation of a wide range of carbo- and heterocyclic compounds [1,2,3,4,5,6]. According to the number of electrons transferred between reaction sites along the reaction course, most cycloadditions are classified as the 4n-π-electron and (4n + 2)-π-electron. The first group of cycloadditions is most often realized via stepwise mechanisms with zwitterionic or biradical intermediates [7,8,9], whereas the second one is predominantly realized according to single-step mechanisms with more or less synchronical transition states [10,11,12]. As the 4n-π-electron processes are classified inter alia [2 + 2] cycloadditions, depending on the nature of addents, the [2 + 2] cycloaddition processes can be considered as the attractive protocol for the synthesis of cyclobutanes [13,14,15], β-lactams [16,17] and other important structures [18].

**Scheme 1:**
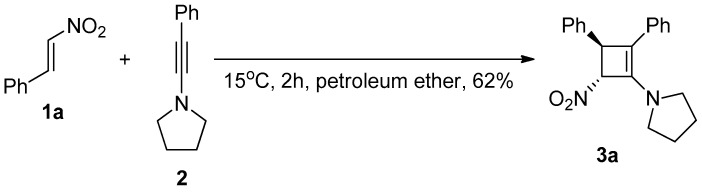
[2 + 2] cycloaddition reaction between (E)-2-phenylnitroethene **1a** and ynamine **2**.

**Scheme 2:**
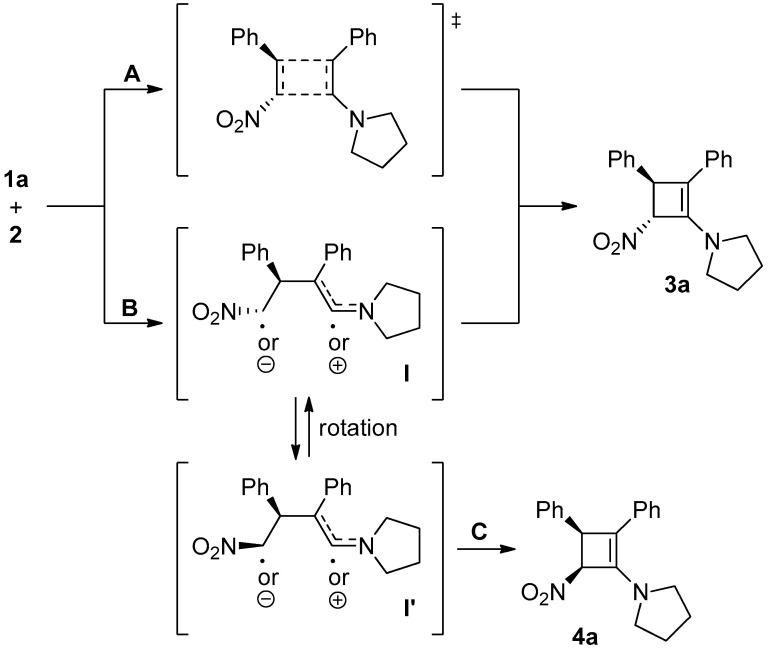
Theoretically possible course of [2 + 2] cycloaddition reaction between (E)-2-phenylnitroethene **1a** and ynamine **2**.

Some years ago, the [2 + 2] cycloaddition reactions between (E)-2-arylnitroethenes and ynamine molecular systems were described as the methodology for the preparation of cyclobutene analogs (Scheme 1) [19,20]. The molecular mechanism of this reaction is, however, very mysterious. Firstly, only one cycloaddition product characterized by the full retention of primary substituent configuration was isolated and identified. It should be noted at this point that in the course of stepwise cycloadditions, in the mass of products, two types of adducts are generally observed—products with the retention of mutual substituent configuration and products with different mutual substituent configuration [21,22,23]. This is a consequence of the rotation around the single bond in the framework of the biradical or zwitterionic intermediate. The full retention of the configuration of substituents is generally observed in the case of single-step (4n + 2)-π-electron cycloadditions [24,25,26,27]. This suggests that the title reaction can exhibit the nature of the rare single-step [2 + 2] cycloadditions. On the other hand, if the discussed reaction is realized via stepwise mechanism, why non-stereospecific products are not observed? The next question is, does the acyclic intermediate along the reaction path exhibit the nature of the biradical or zwitterion (Scheme 2)? Lastly, what is the mechanism of electron density redistribution from reactants to products? In the second part of the XX century, the term “concerted mechanism” was very popular, with ideal circular reorganization of the electron density [28,29,30]. According to the actual state of the knowledge, this point of view was, however, completely undermined. It was confirmed without any doubt that most of the processes defined earlier as “concerted” or “pericyclic” are, in reality, realized via a complicated sequence of phases of the displacement of electron density [31,32,33,34,35]. For the discussed problems, the Molecular Electron Density Theory (MEDT) has become a very good tool and has been successfully applied to the explanation of the selectivity and molecular mechanism of many different type of pericyclic processes, including cycloaddition reactions [36,37,38,39,40]. This theory states that the energy cost associated with the reorganization of the molecular electron density along a reaction path is what determines the chemical reactivity [41].

So, the mechanistic aspects of the title process require a comprehensive exploration. We decide to shed light on this problem using the results of our Density Functional Theory (DFT) quantumchemical calculations. Within these studies, we performed (i) an analysis of the global and local electronic interactions in the framework of the Conceptual Density Functional Theory (CDFT) [42] and analysis of the electronic structure and Electron Localization Function (ELF) [43] topological analysis of ynamine **2**, (ii) a full exploration of the practice reaction channel, including localization and verification of all critical structures and (iii) an interpretation of the Bonding Evolution Theory (BET) [44] model for the representative reaction path. Our studies were realized for the reaction with the participation of the homogenous reaction series of (E)-2-arylnitroethenes **1a**–**c**, characterized by different type of substituents at the phenyl ring (Table 1).

## 2. Results and Discussion

**Figure 1:**
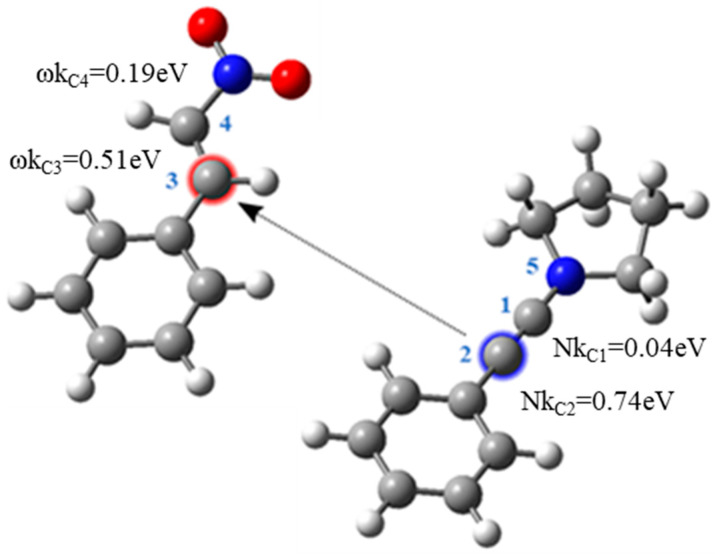
The most electrophilic centers in the structure of **1a** and the most nucleophilic in **2**.

Within the initial step of our research, we decided to shed light on the nature of global and local interactions between considered reaction components. The analogous approach was recently used for the interpretation of different type of bimolecular organic processes, including cycloaddition reactions [36,37,38,46]. Electronic properties of 2-arylnitroethenes were discussed in detail very recently [47]. It was found that all considered nitroalkenes should be treated as strong electrophiles (ω = 2.15–3.70 eV), characterized by the electrophilically activated 2 position of the nitrovinyl moiety. On the other hand, the electronic properties of the ynamine **2** had not yet been explored and described. We found that this molecule is characterized by a very low value of the global electrophilicity descriptor (0.43 eV). So, it should be classified as a marginal electrophilic (nucleophilic) agent [48,49,50]. Its global nucleophilicity index is equal to 2.23 eV. This reactivity is, however, localized, not symmetrical, on the ethynyl moiety. In particular, the local nucleophilicity on the carbon atom connected with the phenyl ring (C2) is equal 0.74 eV (Figure 1), whereas the analogous parameter for the carbon atom connected with the pyrrolidine ring (C1) is only equal to 0.04 eV. So, the reactivity of the ynamine molecular system should be determined by the attack on the more activated center at the C2 carbon atom. The interatomic interactions of this center with the more electrophilic center from the nitroalkene stimulate the formation of the adduct with the geminal orientation of two phenyl rings (**3a**). This prediction correlates excellently with the experimentally observed regioselectivity [19,20].

**Figure 2:**
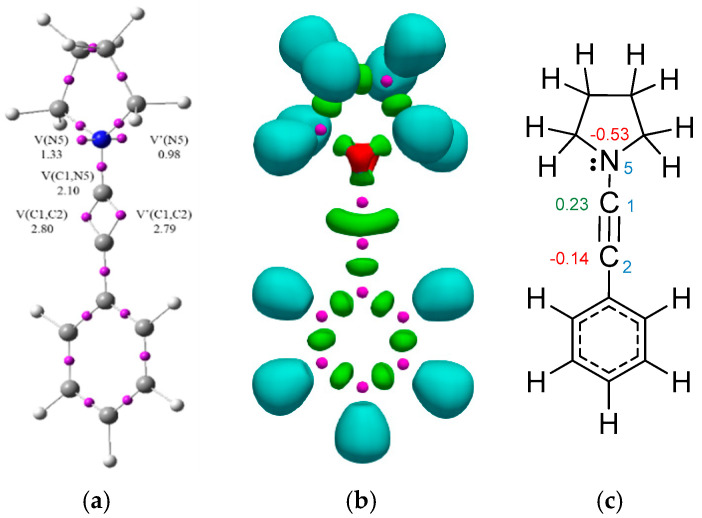
(**a**) ELF basin attractor positions, together with the most representative valence basin populations; (**b**) ELF localization domains of ynamine **2**, represented at an isosurface value of ELF = 0.80; (**c**) Lewis-like structure of **2** together with the natural atomic charges, obtained through an NPA. Negative charges are colored in red, and positive charges are colored in green. Natural atomic charges are given in average number of electrons, e.

In addition to this analysis, we also performed the exploration of the electronic structure of the ynamine **2** based on the ELF [43] approach. In the most important region, ELF analysis of ynamine **2** shows the presence of three disynaptic basins, V(C1,C2), V′(C1,C2) and V(C1,N5), integrating 2.80 e, 2.79 e and 2.10 e, respectively. Two of the disynaptic basins, V(C1,C2) and V′(C1,C2), are associated with the C1≡C2 triple bond, and the V(C1,N5) disynaptic basin with C1-N5 single bond (Figure 2). Two monosynaptic basins at N5 nitrogen, V(N5) and V′(N5), integrating 1.33 e and 0.98 e, respectively, indicate the presence of an N5 lone pair in ynamine **2**.

The natural atomic charges, obtained through the Natural Population Analysis (NPA) [51], are presented in Figure 2c. Based on the NPA of **2**, we can distinguish that the C2 and N5 atoms accumulate the negative charges −0.14 e and −0.53 e, respectively. In turn, the C1 atom has a positive charge, 0.23 e.

Within the second step of our study, we performed the exploration of the reaction profiles. This analysis was started from the parent reaction between nitroalkene **1a** and **2**. The wb97xd/6-311 + G(d) (PCM) calculations show that the nature of the enthalpy profile of this reaction is not typical for single-step cycloaddition. In particular, between valleys of substrates and product, four critical points were detected. The full optimization and vibrational analysis of these structures sheds light on the molecular mechanism of considered transformation

The interactions between addents within the initial reaction phase lead to the formation of the pre-reaction complex **MCB**. This process is realized as the barrier-less transformation and is associated with the reduction of the enthalpy of the reaction system by 11.5 kcal/mol (Table 2). It should be underlined, however, that the great reduction of the entropy of the reaction system determines the positive value of the Gibbs free energy of the formation of the **MCB**. In consequence, at the r.t., this structure cannot be treated as the relatively stable intermediate. From the structural point of view, the **MCB** exhibits the nature of a two-planar complex, with two clearly specified substructures derived from addent molecules. Both substructures adopt the orientation, determining further conversion along the reaction coordinate. So, formally, it can be considered as the orientation complex. Within the **MCB**, the distances between considered substructures are about equal, 3.2–3.4 Å (Table 3). In consequence, the key interatomic distances C2-C3 and C4-C1 exist beyond the range typical for new bonds in transition states [52,53,54,55]. At the same time, any charge transfer between substructures is not observed (GEDT = 0.00 e). Therefore, the localized molecular complex should not be classified as the charge–transfer complex (CT) [56,57,58].

**Figure 3:**
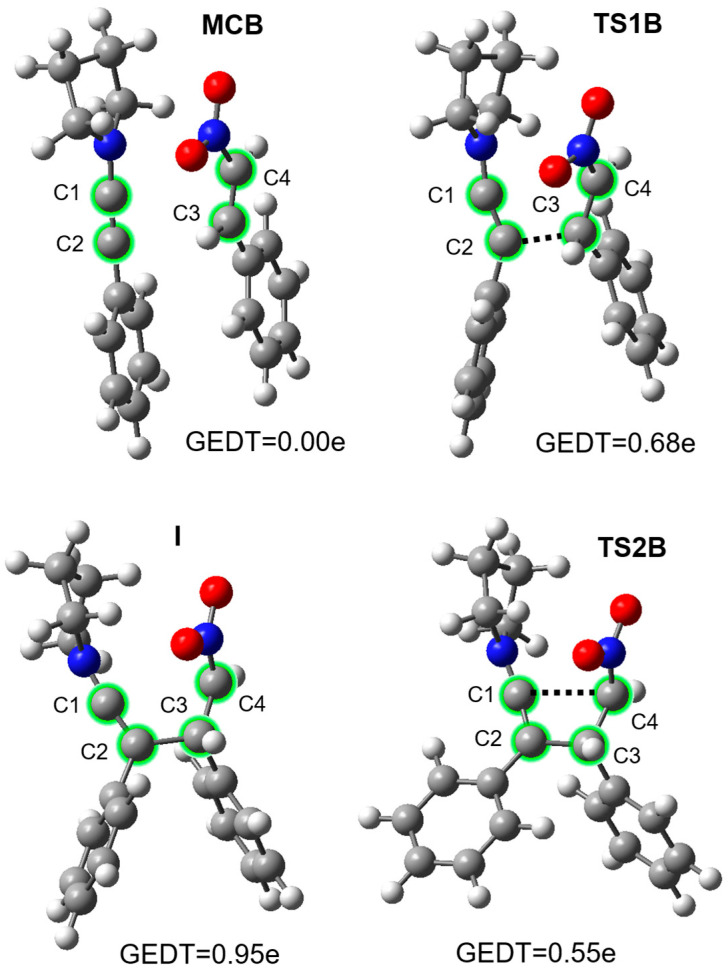
Wb97xd/6-311 + G(d) (PCM) optimized geometries for the **MCB**, **TS1B**, **I** and **TS2B** associated with the [2 + 2] cycloaddition reaction between (E)-2-phenylonitroethene **1a** and ynamine **2**.

The further conversion of the pre-reaction complex leads directly to the point of the transition state. This process is associated with the increasing of the enthalpy of the reaction system by 3.8 kcal/mol. The relatively low value of the enthalpy of the activation is typical for bimolecular processes with polar acceleration [59,60]. This polarity is confirmed by the high value of the GEDT descriptor (0.68 e). Next, according to the direction of the reorganization of the electron density, this transformation should be classified as a Forward Electron Density Flux (FEDF) process [61]. Within the localized transition state, only one new σ-bond is formed between C2 and C3 atoms. So, the localized structure should be not associated with the hypothetical single-step mechanism along the path A but with the competitive stepwise mechanism along the path **B** (Scheme 2). The transitional nature of the localized **TS1B** transition state is confirmed by the vibrational analysis (one imaginary frequency) and the IRC calculations. The second part of the calculations confirms the direct connectivity between **TS1B** with the **MCB** structure and the second reaction intermediate (**I**). The optimized **I** intermediate is characterized by the completed bond C2-C3 (Figure 3). Additionally, the high value of the GEDT (0.95 e) can suggest the non-biradical and probably zwitterionic nature of the intermediate. On the other hand, the open-shell singlet state uWb97xd calculations assign to this structure the enthalpy of the formation, lower by 8.1 kcal/mol than in the case of analogous Wb97xd computations. This indicates that a radical center is available in the analyzed molecular segment. So, at this stage, we can assume that it is a pseudoradical structure (see the further BET analyses). The further conversion of **I** is possible via two independent reaction ways. The first possibility is associated with the creation of the new σ-bond C4-C1. This transformation is realized via the transition state **TS2B**. The activation barrier (ΔG) connected with the formation of this transition state is equal to only 0.2 kcal/mol. So, this process should be considered as very fast under the reaction conditions. The IRC calculations connect the transition state **TS2B** with the valley of the intermediate **I** and with the valley of the expected reaction product **3a**. Within this adduct, both bonds necessary for the formation of the four-membered ring are completed. The conversion of the reaction system to this product is very attractive from the thermodynamic point of view. Alternatively, the **I** structure can convert to the stereoisomeric intermediate in consequence of the intramolecular rotation around the C3-C4 bond. This transformation is, however, not preferred from the kinetic point of view, because respective barrier of the activation is 2.8 kcal/mol higher than in the case of mentioned above cyclization process. This excludes, rather, the possibility of the isomerization of intermediate in the conditions of the competition with the cyclization. Therefore, the considered cycloaddition process is realized with the retention of the primary configuration of substituents in the nitroalkene structure, independent of the stepwise nature of the cycloaddition (the stepwise mechanism generally allows the formation of the non-stereospecific cycloadducts) [19,20].

The introduction of more polar solvent to the reaction environment does not change the nature of the reaction profiles. The quantitative descriptions of critical points are corrected, however, in some ranges. In particular, changes of the energy of the reaction system connected to the formation of **MCs** are slightly lower. At the same time, the respective barriers of the activation are reduced. These changes are, however, not significant and should be not enforced to dramatically change the reaction conditions. This is interesting, because in the case of postulate zwitterionic mechanism, the great influence of the polarity of the solvent on the reaction course should be expected. The changes of the energetical description of critical points are accompanied by some corrections of geometrical parameters of localized critical structures. These changes are, however, not sufficient to enforce an alternate type of reaction mechanism.

Next, the analogous processes with the participation of nitroalkenes **1b**,**c** were explored. It was found that the nature of the reaction profiles is an analogous process with the participation of the 2-phenylnitroethene **1a**. As can be expected in light of the analysis of reactivity descriptors, the presence of the electron donating group (EDG) substituent stimulates higher barriers of the activation, whereas the presence of the electron withdrawing group (EWG) substituents favors the lower activation barriers. The substituent effects have, however, not qualitatively influenced the cycloaddition mechanism. For the analyzed range of electrophilic agents, this mechanism can be treated as general.

**Figure 4:**
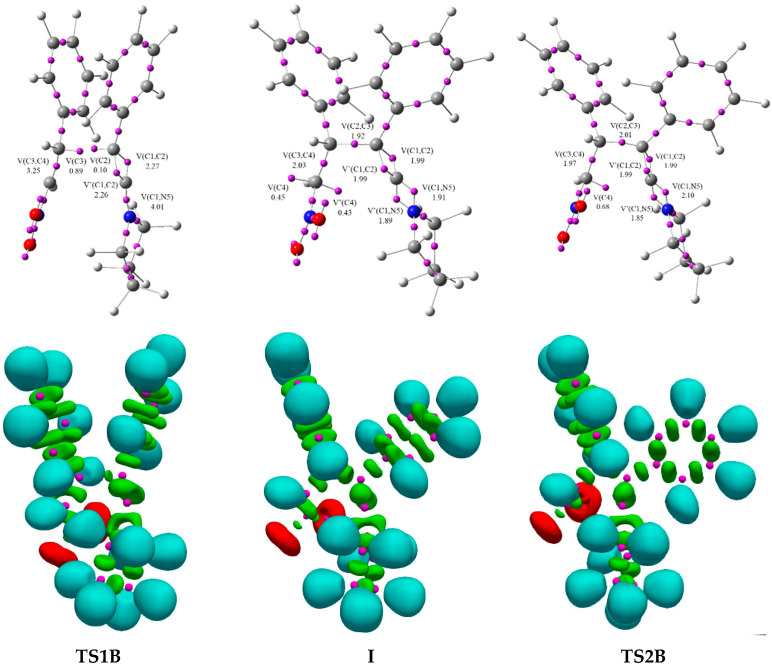
The most significant attractor positions of the ELF valence basins of the structures **TS1B**, **I** and **TS2B** participating in the C2-C3 and C4-C1 single-bond formations participating in [2 + 2] cycloaddition reaction between nitroalkene **1a** and ynamine **2**. The electron populations, in average number of electrons, are given in e.

**Figure 5:**
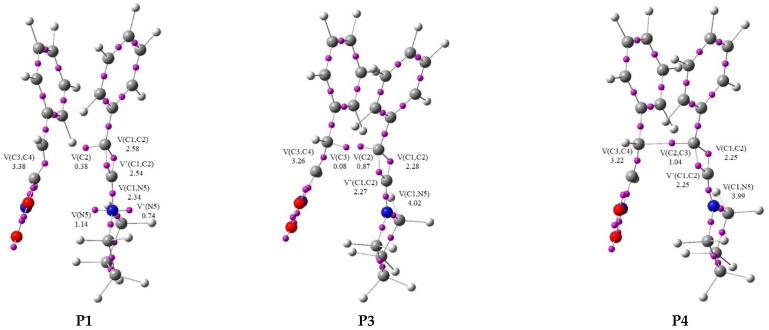

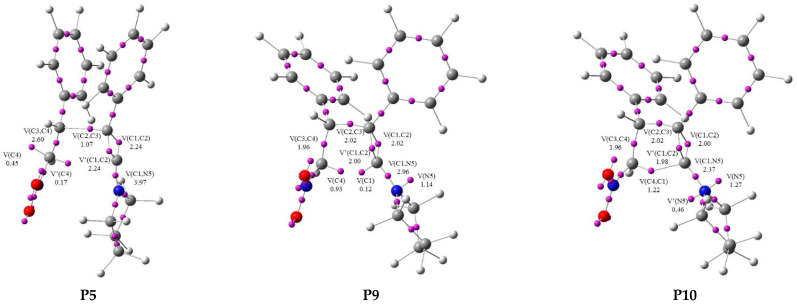
The most significant attractor positions of the ELF valence basins of the structures **P1**, **P4**-**P5**, **P9** and **P10** participating in the C2-C3 and C4-C1 single bond formation participating in [2 + 2] cycloaddition reaction between (E)-2-phenylonitroethene **1a** and ynamine **2**. The electron populations, in average number of electrons, are given in e.

Lastly, the question of the detailed description of redistribution of the electron density along the reaction coordinate was evaluated on the basis of BET [44] analysis. The populations, among other relevant parameters, of the most significant ELF valence basins of the selected points of the IRC, Pi, defining the different topological phases are gathered in Table 4, while the ELF localization domains of the most significant attractor positions of the ELF valence basins of the structures **TS1B**, **I** and **TS2B** and their attractor positions for the points involved in the bond formation processes are shown in Figure 4 and Figure 5, respectively.

**Scheme 3:**
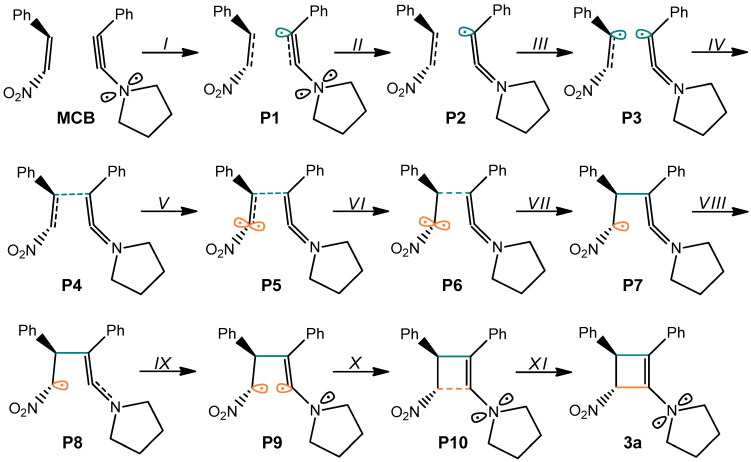
The molecular mechanism of the [2 + 2] cycloaddition reaction between (E)-2-phenylonitroethene **1a** and ynamine **2** by Lewis-like structures resulting from the topological analysis of the ELF along the reaction path.

The *Phase I*, 2.68 Å ≥ d (C2-C3) > 2.42 Å and 3.08 Å ≥ d (C4-C1) > 2.99 Å, begins at the molecular complex (**MCB**), which is the minimum in the reaction path connecting the separated reagents, **1a** and **2**. The ELF topological analysis of **MCB** reflects the ELF image of the individual reactants **1a** and **2**. The two disynaptic basins, V(C3,C4) and V′(C3,C4), present in **1a** in the **MCB** are combined into the new V(C3,C4) disynaptic basin integrating 3.41 e (see Table 4 and Figure 5). This disynaptic basin is associated with a double bond between atoms C3 and C4 (Scheme 3).

At **P1**, *Phase II*, 2.42 Å ≥ d (C2-C3) > 2.13 Å and 2.99 Å ≥ d (C4-C1) > 2.93 Å, begins, in which the first significant changes along the reaction path take place. At this point, we observed the formation of the new monosynaptic basin V(C2) integrating 0.38 e. This topological change is related to formation of a *pseudoradical* center at the C2 carbon atom (Figure 5).

*Phase III*, 2.13 Å ≥ d (C2-C3) > 2.10 Å and d (C4-C1) = 2.93 Å, begins at **P2**. In this phase, we observed the disappearance of two monosynaptic basins, V(N5) and V′(N5). The population of the V(C1,N5) disynaptic basin considerably increases, reaching 4.02 e at the end of the phase.

At *Phase IV*, 2.10 Å ≥ d (C2-C3) > 2.06 Å and 2.93 Å ≥ d (C4-C1) > 2.92 Å, which begins at **P3**, the next important topological change along the reaction takes place. At this point, we observed the formation of the new V(C3) monosynaptic basin, integrating 0.08 e. This topological change is associated with the formation of a new pseudoradical center at the C3 carbon atom (Figure 5). The first transition state **TS1B** of the analyzed reaction is found in this phase (d (C2-C3) = 2.084 Å and d (C4-C1) = 2.924 Å). The ELF picture of **TS1B** presents only slight variations in the ELF basin populations with respect to the topological features of **P3** (see Figure 4).

In *Phase V*, 2.06 Å ≥ d (C2-C3) > 2.04 Å and d (C4-C1) = 2.92 Å, which begins in **P4**, we observed the formation of the first new C2-C3 single bond. This bond is created by combining the two monosynaptic basins V(C2) and V(C3) into one new disynaptic basin, V(C2,C3), integrating 1.04 e. The C2-C3 single bond is formed at C2-C3 length d (C2-C3) = 2.057 Å (see Table 4 and Figure 5).

At *Phase VI*, 2.04 Å ≥ d (C2-C3) > 1.86 Å and 2.92 Å ≥ d (C4-C1) > 2.88 Å, which starts at **P5**, we observed the next significant topological change. At this phase, two new monosynaptic basins, V(C4) and V′(C4), are created, integrating 0.45 e and 0.17 e, respectively. This topological change is related to the formation of a *pseudoradical* center at C4 carbon atom. The formation of these two monosynaptic basins is also associated with the depopulation of the V(C3,C4) bonding region. The population of the V(C3,C4) disynaptic basin is 2.60 e (Table 4).

At *Phase VII*, 1.86 Å ≥ d (C2-C3) > 1.52 Å and 2.88 Å ≥ d (C4-C1) > 2.39 Å, which begins at **P6**, we notice the division of the V(C1,N5) disynaptic basin, present in previous phase, for two new V(C1,N5) and V′(C1,N5) disynaptic basins integrating 2.00 e and 1.87 e, respectively. The intermediate **I** of the analyzed reaction is found in this phase (d (C2-C3) = 1.542 Å and d (C4-C1) = 2.758 Å). The ELF picture of **I** shows slight changes in ELF valence basin populations compared to point **P6** (see Figure 4). A thorough BET analysis of **I** allowed for a detailed analysis of the electronic structure of **I**. The presence of two V(C4) and V′(C4) monosynaptic basins at the C4 carbon atom allowed (with the full accordance of the previous discussion) for the classification of this compound as a pseudoradical structure.

At **P7**, *Phase VIII* begins, 1.52 Å ≥ d (C2-C3) > 1.51 Å and 2.39 Å ≥ d (C4C1) > 2.25 Å, in which we observed the disappearance of the V′(C4) monosynaptic basin. The disappearance of the V′(C4) monosynaptic basin causes an increase in the population of the V(C4) monosynaptic basin to 0.66e. The second transition state, **TS2B**, of the tested reaction is in this phase (d (C2-C3) = 1.514 Å and d (C4-C1) = 2.301 Å). The most significant attractor positions of the ELF valence basins of the structure **TS2B** we can find in Figure 4. The attractor positions of the **TS2B** differ slightly from the values presented in point **P7**.

In *Phase IX*, d (C2-C3) = 1.51 Å and 2.25 Å ≥ d (C4-C1) > 2.10 Å, which begins at **P8**, we observed the formation of a new disynaptic basin, V(C1,N5), integrating 3.98e, through the connection of two disynaptic basins, V(C1,N5) and V′(C1,N5), present in the previous phase.

At *Phase X*, d (C2-C3) = 1.51 Å and 2.10 Å ≥ d (C4-C1) > 2.06 Å, which begins at **P9**, we observed the formation of two new monosynaptic basins, V(C1) and V(N5), integrating 0.12e and 1.14e, respectively. Formation of a new V(N5) monosynaptic basin causes a decrease in the value of V(C1,N5) disynaptic basin. The formation of a new V(C1) monosynaptic basin is associated with the formation of a *pseudoradical* center at C1 carbon atom.

The last *Phase XI*, d (C2-C3) = 1.51 Å and 2.06 Å ≥ d (C4-C1) > 1.62 Å is located between **P10** and **3a**. In this phase, we observed the last important topological change. We observed formation of a new disynaptic basin, V(C4,C1), with a population of 1.22 e through the connection of two monosynaptic basins, V(C1) and V(C4). This topological change is related to the formation of a second C4-C1 single bond. This phase is also connected with the creation of a new monosynaptic basin, V′(N5), by the depopulation of the disynaptic basin V(C1,N5) (see Table 4 and Figure 5).

**Figure 6:**
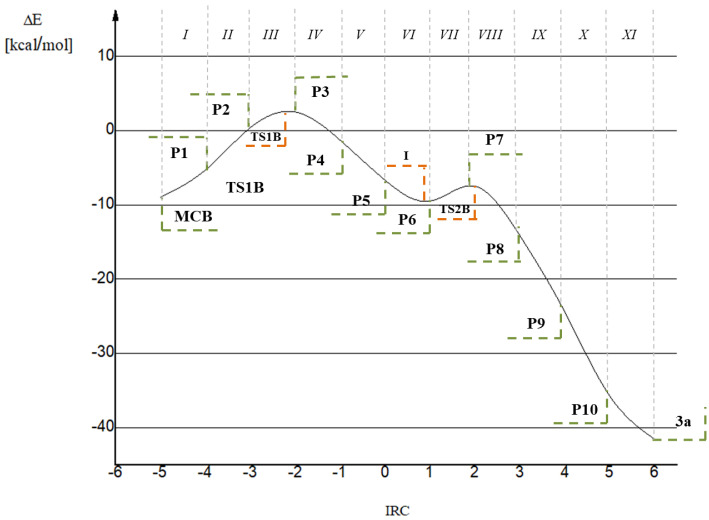
Wb97xd/6-311 + G(d) (PCM) IRC profile, including all points participating in [2 + 2] cycloaddition reaction between (E)-2-phenylonitroethene **1a** and ynamine **2**.

A BET analysis of the model [2 + 2] cycloaddition reaction between (E)-2-phenylonitroethene **1a** and ynamine **2** allow us to conclude that (i) this [2 + 2] cycloaddition reaction takes place through eleven different phases (Figure 6 and Scheme 3); (ii) the formation of the first C2-C3 single bond takes place in Phase V at a C2-C3 distance of 2.057 Å by connection of two monosynaptic basins, V(C2) and V(C3); (iii) the formation of a second C4-C1 single bond begins at the last phase of the reaction path, by connection of two monosynaptic basins, V(C4) and V(C1); (iv) according to BET analysis, we can determine the electronic structure of **I**. The presence of two monosynaptic basins, V(C4) and V′(C4), at the C4 carbon atom allowed for the classification of this compound as a *pseudoradical* structure.

## 3. Computational Details

The computational study was performed using the wb97xd/6-311 + G(d) level of theory with the Gaussian 16 package [62]. The PLGrid infrastructure (“Ares” supercomputer) at the national computing center “Cyfronet” was utilized. All optimized critical points were verified on the basis of the full vibrational analysis. We found that addent molecules, pre-reaction, intermediates and products had positive Hessian matrices, while all optimized transition states exhibited only one negative eigenvalue in their Hessian matrices. For the identification of radical centers within the reaction intermediate, open-shell, singlet state uwb97xd calculations were performed. Recently, the analogous methodology was used for the searching of radical nature of some other intermediates along the way of pericyclic processes [63,64].

Next, intrinsic reaction coordinate (IRC) calculations were performed for all optimized transition states. The obtained IRC trajectories confirmed, without a doubt, the postulated nature of the TSs and their role within the energy profile. The presence of solvents (n-pentane, acetone and nitromethane) in the reaction environment was included using the IEFPCM (Integral Equation Formalism Polarizable Continuum Model) algorithm [65]. Calculations of all critical structures were performed at temperature T = 298 K and pressure *p* = 1 atm.

The global electron density transfer (GEDT) [66,67] within critical structures was estimated using the formula

GEDT = −ΣqA(1)

where qA is the net charge, and the sum is taken over all the atoms of nitroalkene.

Global and local electronic properties of the reactants were estimated using equations recommended by *Parr* and *Domingo* [68,69]. In particular, the electronic chemical potentials (µ) and chemical hardness (η) were evaluated in terms of one-electron energies of the frontier molecular orbitals (HOMO and LUMO) using the following equations:

μ ≈ (E_HOMO_ + E_LUMO_)/2 (2)

η ≈ E_LUMO_ − E_HOMO_ (3)

The values of µ and η were then used to calculate the global electrophilicity index (ω) using the formula

ω = μ^2^/2η (4)

Global nucleophilicity (N) [70] was expressed using the equation

N = E_HOMO_ − E_HOMO(tetracyanoethene)_ (5)

The local electrophilicity (ω_k_) at atom k was calculated by projecting the index ω into any reaction center k in the molecule using *Parr* functions P^+^_k_ [71]:

ω_k_ = P^+^_k_·ω (6)

The local nucleophilicity (N_k_) condensed to atom k was calculated using global nucleophilicity N and Parr functions P^−^_k_ [71] according to the formula

N_k_ = P^−^_k_·N (7)

Indexes of σ-bonds development (l) were calculated according to the formula [72]

(8) $$I_{X-Y}=1-\frac{r_{X-Y}^{TS}-r_{X-Y}^{P}}{r_{X-Y}^{P}}$$

where r^TS^_X−Y_ is the distance between the reaction centers X and Y in the transition structure, and r^P^_X−Y_ is the same distance in the corresponding product.

The ELF [43] studies were performed with the TopMod package 2.0 [73], considering the standard cubical grid of step size of 0.1 Bohr. The bonding changes along corresponding reactions were analyzed, according to the BET [44], by performing the topological analysis of the ELF for 202 nuclear configurations for reaction leading to **I** and 200 nuclear configurations leading to product **3a**. The ELF molecular geometries and basin attractor positions were visualized using the GaussView program 6.1 [74]. ELF localization domains were represented by using the Paraview software (https://www.paraview.org/) at an isovalue of 0.80 a.u [75,76]. Key results are collected in Tables and Supplementary Materials.

## 4. Conclusions

The results of the wb97xd/6-311 + G(d) quantumchemical calculations offer a possibility of the interpretation of the regio- and stereoselectivity and the molecular mechanism of the [2 + 2] cycloaddition reaction between E-2-arylnitroethenes and the ynamine molecular system. It was found that the reaction course is determined by the nucleophilic attack of the activated nucleophilic site of the ynamine molecule to the 2-position of the nitrovinyl moiety. So, the experimentally observed regioselectivity of the reaction is a natural consequence of this attack. The first reaction stage is, however, the formation of the acyclic intermediate. This intermediate can theoretically rotate around the C-C(NO_2_) bond. The cyclization process with the retention of the stereoconfiguration is, however, more favored. In consequence, the full stereospecificity of the cycloaddition process is observed independently of the stepwise nature of the reaction mechanism. Our extended studies of solvent and substituent effects show that the proposed mechanism can be treated as general for some ranges of similar transformations. As an addition, the detailed BET studies were performed for the model process with the participation of the 2-phenylnitroehene. A detailed BET analysis of the [2 + 2] cycloaddition reaction between **1a** and **2** indicates that this process can be described by eleven different phases associated with the change in electron density redistribution. The process begins with the breaking of the double bonds in **1a** and the triple bonds in molecule **2**. Next, we observed the formation of two *pseudoradical* centers at the C2 and C3 atoms. First, the C2-C3 single bond is formed in phase V by the combining of two *pseudoradical* centers. In turn, the formation of a second single bond, C4-C1, begins at the last phase of the reaction path, by connection of two monosynaptic basins, V(C4) and V(C1). Additionally, a BET analysis of intermediate (I) due to the presence of two *pseudoradical* centers on the C4 atom, allows **I** to be classified as a compound with a *pseudoradical* structure. So, in light of the MEDT studies, we propose the new type of the intermediate for [2 + 2] cycloaddition processes, next to the generally known biradicals and zwitterions.

## Figures and Tables

**Table 1:** (E)-2-arylnitroethenes **1a**–**c** considered as partners for reaction with the ynamine **2**.

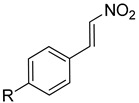	structure	R	σ_R_ [45]
	**1a**	H	0.00
	**1b**	NMe_2_	−0.72
	**1c**	NO_2_	0.78

**Table 2:** Key kinetic and thermodynamic parameters of the cycloaddition reactions between (E)-2-arylnitroethenes **1a**–**c** and ynamine **2** according to the wb97xd/6-311 + G(d) (PCM) calculations (ΔH and ΔG are in kcal/mol; ΔS are in cal/mol·K).

Reaction	Solvent	Transition	ΔH	ΔS	ΔG
**1a** + **2**	n-Pentane	**1a** + **2**→**MCB**	−11.5	−44.9	1.8
		**1a** + **2**→**TS1B**	**3.8**	−**55.2**	**20.3**
		**1a** + **2**→**I**	−1.3	−51.1	13.9
		**1a** + **2**→**TS2B**	−**1.3**	−**51.7**	**14.1**
		**1a** + **2**→**3a**	−44.3	−52.6	−28.6
		**1a** + **2**→**TSrot**	**0.8**	−**53.8**	**16.9**
		**1a** + **2**→**I′**	−0.6	−53.4	15.4
		**1a** + **2**→**TSC**	**0.9**	−**53.5**	**16.8**
		**1a** + **2**→**4a**	−43.7	−55.3	−27.2
	Acetone	**1a** + **2**→**MCB**	−9.6	−44.5	3.6
		**1a** + **2**→**TS1B**	2.0	−53.9	18.1
		**1a** + **2**→**I**	−9.5	−50.5	5.6
		**1a** + **2**→**TS2B**	−7.6	−51.1	7.6
		**1a** + **2**→**3a**	−42.2	−52.5	−26.6
		**1a** + **2**→**TSrot**	−7.3	−53.1	8.5
		**1a** + **2**→**I′**	−8.2	−53.9	7.8
		**1a** + **2**→**TSC**	−4.4	−55.1	12.0
		**1a** + **2**→**4a**	−41.9	−55.2	−25.5
	Nitromethane	**1a** + **2**→**MCB**	−9.5	−44.6	3.8
		**1a** + **2**→**TS1B**	1.9	−54.4	18.1
		**1a** + **2**→**I**	−10.1	−49.2	4.6
		**1a** + **2**→**TS2B**	−8.1	−51.4	7.3
		**1a** + **2**→**3a**	−42.2	−51.5	−26.8
		**1a** + **2**→**TSrot**	−8.0	−52.2	7.5
		**1a** + **2**→**I′**	−8.8	−53.1	7.0
		**1a** + **2**→**TSC**	−4.9	−55.0	11.5
		**1a** + **2**→**4a**	−41.9	−55.2	−25.4
**1b** + **2**	n-Pentane	**1b** + **2**→**MCB**	−12.7	−45.9	0.9
		**1b** + **2**→**TS1B**	6.0	−55.3	22.4
		**1b** + **2**→**I**	1.5	−53.3	17.4
		**1b** + **2**→**TS2B**	1.3	−54.7	17.6
		**1b** + **2**→**3b**	−41.2	−54.0	−25.1
**1c** + **2**	n-Pentane	**1c** + **2**→**MCB**	−14.5	−47.7	−0.3
		**1c** + **2**→**TS1B**	1.1	−54.4	17.3
		**1c** + **2**→**I**	−4.5	−53.6	11.4
		**1c** + **2**→**TS2B**	−4.1	−54.0	12.0
		**1c** + **2**→**3c**	−46.8	−53.3	−31.0

**Table 3:** Key parameters of critical structures for the cycloaddition reactions between (E)-2-arylnitroethenes **1a**–**c** and ynamine **2** according to the wb97xd/6-311 + G(d) (PCM) calculations.

Reaction	Solvent	Structure	Interatomic Distances r [Å]				Bond Development l		GEDT
			C1-C2	C2-C3	C3-C4	C4-C1	C2-C3	C4-C1	[e]
**1** + **2a**	n-Pentane	**2**	1.210						
		**1a**			1.332				
		**MCB**	1.211	3.209	1.333	3.336			0.00
		**TS1B**	1.251	1.975	1.404	2.905	0.737		0.68
		**I**	1.288	1.564	1.489	2.756			0.95
		**TS2B**	1.308	1.525	1.506	2.426		0.384	0.55
		**3a**	1.356	1.520	1.571	1.501			
		**TSrot**	1.288	1.554	1.490	2.757			0.75
		**I′**	1.298	1.541	1.541	2.688			0.52
		**TSC**	1.308	1.521	1.512	2.418		0.388	0.51
		**4a**	1.358	1.520	1.580	1.499			
	Acetone	**2**	1.213						
		**1a**			1.334				
		**MCB**	1.211	3.185	1.335	3.295			0.00
		**TS1B**	1.245	2.077	1.397	2.923	0.654		0.64
		**I**	1.294	1.543	1.496	2.759			0.80
		**TS2B**	1.312	1.515	1.511	2.312		0.460	0.68
		**3a**	1.358	1.521	1.571	1.502			
		**TSrot**	1.295	1.543	1.497	2.720			0.60
		**I′**	1.296	1.532	1.502	2.797			0.62
		**TSC**	1.308	1.521	1.512	2.418		0.388	0.61
		**4a**	1.361	1.520	1.580	1.500			
	Nitromethane	**2**	1.213						
		**1a**			1.334				
		**MCB**	1.211	3.182	1.335	3.293			0.00
		**TS1B**	1.245	2.084	1.396	2.924	0.649		0.64
		**I**	1.294	1.542	1.496	2.758			0.80
		**TS2B**	1.312	1.514	1.512	2.301		0.468	0.71
		**3a**	1.358	1.521	1.571	1.502			
		**TSrot**	1.295	1.542	1.497	2.722			0.60
		**I′**	1.296	1.532	1.502	2.800			0.63
		**TSC**	1.313	1.513	1.517	2.277		0.481	0.61
		**4a**	1.361	1.520	1.580	1.500			
**1** + **2b**	n-Pentane	**1b**			1.338				
		**MCB**	1.211	3.442	1.340	3.537			0.00
		**TS1B**	1.252	1.960	1.410	2.900	0.749		0.69
		**I**	1.288	1.567	1.490	2.733			1.07
		**TS2B**	1.307	1.528	1.506	2.450		0.367	0.50
		**3b**	1.356	1.521	1.574	1.501			
**1** + **2c**	n-Pentane	**1c**			1.329				
		**MCB**	1.211	3.425	1.330	3.410			0.00
		**TS1B**	1.252	1.976	1.402	2.917	0.730		0.73
		**I**	1.294	1.556	1.492	2.715			0.64
		**TS2B**	1.309	1.524	1.506	2.424		0.386	0.39
		**3c**	1.357	1.519	1.569	1.501			

**Table 4:** ELF valence basin populations and distances of the forming bonds based on wb97xd/6-311 + G(d) (PCM) calculations of the IRC structures, **MCB**—**3a**, defining the eleven different phases characterizing the molecular mechanism of the [2 + 2] cycloaddition reactions between **1a** and **2**. Distances are given in angstroms, Å, relative energies in kcal·mol^−1^, and electron populations in average number of electrons, e.

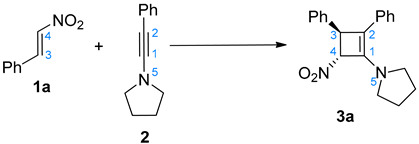														
Points	1a	2	MCB	P1	P2	P3	P4	P5	P6	P7	P8	P9	P10	3a
Phases			*I*	*II*	*III*	*IV*	*V*	*VI*	*VII*	*VIII*	*IX*	*X*	*XI*	
d(C2–C3)			2.681	2.417	2.125	2.098	2.057	2.044	1.863	1.519	1.513	1.511	1.511	1.514
d(C4–C1)			3.076	2.994	2.931	2.926	2.919	2.917	2.879	2.389	2.247	2.103	2.055	1.622
ΔE ^a^			−9.0	−5.3	0.4	2.5	−1.3	−6.7	−9.5	−7.5	−14.2	−25.3	−36.8	−41.6
V(C1,C2)		2.80	2.87	2.58	2.30	2.28	2.25	2.24	2.15	1.98	1.99	2.02	2.00	1.85
V′(C1,C2)		2.79	2.65	2.54	2.29	2.27	2.25	2.24	2.14	1.98	1.99	2.00	1.98	1.82
V(C1)												0.12		
V(C2)				0.38	0.84	0.87								
V(C3)						0.08								
V(C2,C3)							1.04	1.07	1.40	2.00	2.02	2.02	2.02	1.99
V(C3,C4)	1.74		3.41	3.38	3.34	3.26	3.22	2.60	2.31	1.98	1.97	1.96	1.96	1.89
V′(C3,C4)	1.74													
V(C4)								0.45	0.55	0.66	0.76	0.93		
V′(C4)								0.17	0.25					
V(C4,C1)													1.22	1.95
V(C1,N5)		2.10	2.22	2.34	4.03	4.02	3.99	3.97	2.00	2.00	3.98	2.96	2.37	1.84
V′(C1,N5)									1.87	1.88				
V(N5)		1.33	1.34	1.14								1.14	1.27	1.87
V′(N5)		0.98	0.74	0.74									0.46	0.65

^a^ Relative to the separated reagents **1a** and **2**.

## Data Availability

The original contributions presented in this study are included in the article. Further inquiries can be directed to the corresponding author.

## Supplementary Materials

The following supporting information can be downloaded at: https://www.mdpi.com/article/10.3390/molecules30112410/s1, Figure S1. Other attractor positions of the ELF valence basins of the structures **1a**, **MCB**, **P2**, **P6**-**P8** and **3a** participating in the C2-C3 and C4-C1 single bonds formation in [2 + 2] cycloaddition reaction between (E)-2-phenylonitroethene **1a** and ynamine **2**. The electron populations, in average number of electrons, are given in e.
