# Supplementary material for: The Puzzle of the New Type of Intermediate in the Course of [2 + 2] Cycloaddition with the Participation of Conjugated Nitroalkenes: MEDT Computational Study
Source: Molecules. 2025 May 30;30(11):2410. doi: 10.3390/molecules30112410 (PMC12156064; doi:10.3390/molecules30112410)
Supplement: Supplementary file 1 [file molecules-30-02410-s001.zip › molecules-3655848-supplementary.pdf]

Supplementary Material

The puzzle of the new type of the intermediate in the course of the [2+2] cycloaddition with the participation of conjugated nitroalkenes: MEDT computational study

Radomir Jasiński [1,*] and Agnieszka Kącka-Zych [1,*]

[1] Department of Organic Chemistry and Technology, Cracow University of Technology Warszawska 24, 31-155 Cracow, Poland,;

* Correspondence: radomir.jasinski@pk.edu.pl; agnieszka.kacka-zych@pk.edu.pl

Index

S2 **Figure S1.** Other attractor positions of the ELF valence basins of the structures **1a**, **MCB**, **P2**, **P6**-**P8** and **3a** participating in the C2-C3 and C4-C1 single bonds formation in [2+2] cycloaddition reaction between (E)-2-phenylonitroethene **1a** and ynamine **2**. The electron populations, in average number of electrons, are given in e.

Figure S1. Other attractor positions of the ELF valence basins of the structures **1a**, **MCB**, **P2**, **P6-P8** and **3a** participating in the C2-C3 and C4-C1 single bonds formation in [2+2] cycloaddition reaction between (E)-2-phenylonitroethene **1a** and ynamine **2**. The electron populations, in average number of electrons, are given in e.

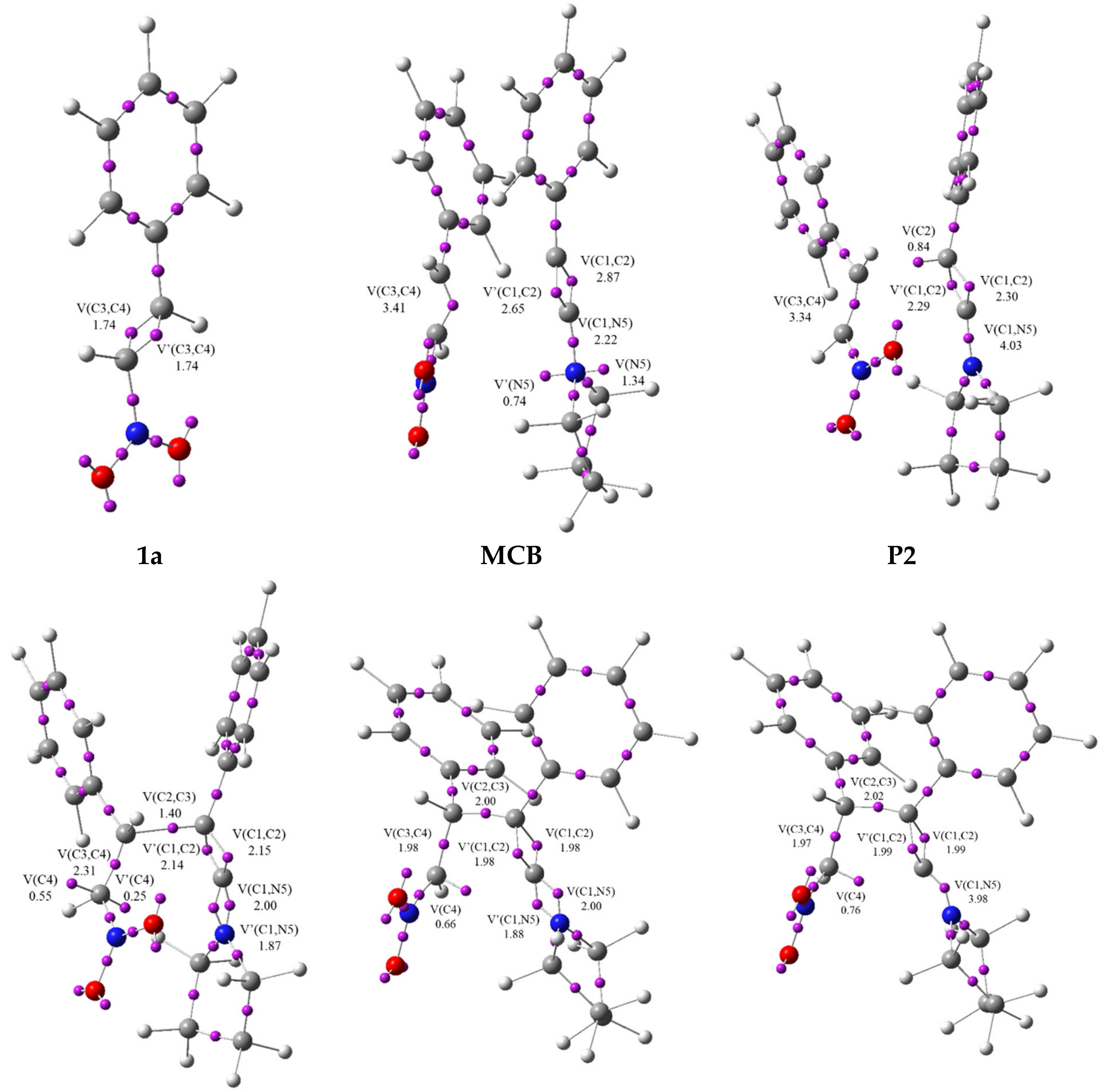

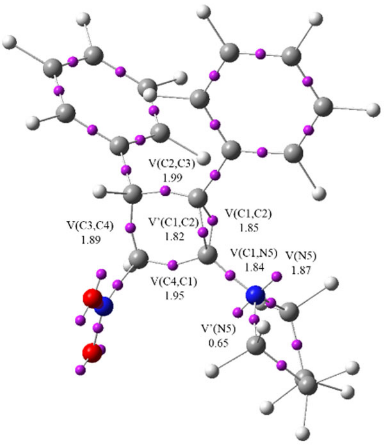
V(C2,C3)
1.99
V(C3,C4)
1.89
V'(C1,C2)
1.82
V(C1,C2)
1.85
V(C1,N5)
1.84
V(N5)
1.87
V(C4,C1)
1.95
V'(N5)
0.65

3a
